# Assessing deep learning reconstruction for faster prostate MRI: visual vs. diagnostic performance metrics

**DOI:** 10.1007/s00330-024-10771-y

**Published:** 2024-05-09

**Authors:** Quintin van Lohuizen, Christian Roest, Frank F. J. Simonis, Stefan J. Fransen, Thomas C. Kwee, Derya Yakar, Henkjan Huisman

**Affiliations:** 1 University Medical Centre Groningen, Hanzeplein 1, 9713 GZ Groningen, The Netherlands; 2 University of Twente, Drienerlolaan 5, 7522 NB Enschede, The Netherlands; 3 Netherlands Cancer Institute, Plesmanlaan 121, 1066 CX Amsterdam, The Netherlands; 4 Radboud University Medical Centre, Geert Grooteplein Zuid 10, 6525 GA Nijmegen, The Netherlands; 5 Norwegian University of Science and Technology, Høgskoleringen 1, 7034 Trondheim, Norway

**Keywords:** Magnetic resonance imaging, Deep learning, Cancer of prostate, Diagnosis (computer-assisted), Image analysis (computer-assisted)

## Abstract

**Objective:**

Deep learning (DL) MRI reconstruction enables fast scan acquisition with good visual quality, but the diagnostic impact is often not assessed because of large reader study requirements. This study used existing diagnostic DL to assess the diagnostic quality of reconstructed images.

**Materials and methods:**

A retrospective multisite study of 1535 patients assessed biparametric prostate MRI between 2016 and 2020. Likely clinically significant prostate cancer (csPCa) lesions (PI-RADS $\ge$ 4) were delineated by expert radiologists. T2-weighted scans were retrospectively undersampled, simulating accelerated protocols. DL reconstruction (DLRecon) and diagnostic DL detection (DLDetect) were developed. The effect on the partial area under (pAUC), the Free-Response Operating Characteristic (FROC) curve, and the structural similarity (SSIM) were compared as metrics for diagnostic and visual quality, respectively. DLDetect was validated with a reader concordance analysis. Statistical analysis included Wilcoxon, permutation, and Cohen’s kappa tests for visual quality, diagnostic performance, and reader concordance.

**Results:**

DLRecon improved visual quality at 4- and 8-fold (R4, R8) subsampling rates, with SSIM (range: −1 to 1) improved to 0.78 ± 0.02 (*p* < 0.001) and 0.67 ± 0.03 (*p* < 0.001) from 0.68 ± 0.03 and 0.51 ± 0.03, respectively. However, diagnostic performance at R4 showed a pAUC FROC of 1.33 (CI 1.28–1.39) for DL and 1.29 (CI 1.23–1.35) for naive reconstructions, both significantly lower than fully sampled pAUC of 1.58 (DL: *p* = 0.024, naïve: *p* = 0.02). Similar trends were noted for R8.

**Conclusion:**

DL reconstruction produces visually appealing images but may reduce diagnostic accuracy. Incorporating diagnostic AI into the assessment framework offers a clinically relevant metric essential for adopting reconstruction models into clinical practice.

**Clinical relevance statement:**

In clinical settings, caution is warranted when using DL reconstruction for MRI scans. While it recovered visual quality, it failed to match the prostate cancer detection rates observed in scans not subjected to acceleration and DL reconstruction.

## Key Points

- *Increasing demand for prostate MRI requires ongoing optimisation of MR techniques to maximise efficiency while maintaining accuracy.*
- *Deep learning reconstruction models may reduce diagnostic performance at 4- and 8-fold subsampling rates.*
- *Automated quantifiable metrics focused on diagnostic quality are recommended when evaluating DL reconstruction methods in MRI.*

## Introduction

Magnetic resonance imaging (MRI) has experienced significant growth in clinical use, evidenced by a more than threefold increase in procedures from 1997 to 2006 [1]. This upward trend continues, especially with the ongoing exploration into MRI’s role in prostate cancer screening [2]. This expansion highlights the need for faster, more efficient MRI techniques in response to the growing demand. Deep learning reconstruction (DLRecon) offers a promising avenue for accelerating MRI acquisitions and increasing capacity. However, these models are typically trained and validated using conventional image quality metrics (ImagQMs), such as the structural similarity (SSIM) and the peak signal-to-noise ratio (PSNR). Unfortunately, these ImagQMs focus solely on visual quality and poorly align with visual quality assessments by radiologists [3]. However, it is vital to note that visual quality, whether assessed computationally or by a radiologist, does not address the primary goal of diagnostic imaging: disease detection and characterisation.

A concern with optimising DLRecon models based on ImagQMs is the introduction of ‘hallucinatory effects’—artificial image artifacts or distortions not present in the original data [4]. These hallucinations can significantly compromise the diagnostic integrity of the images, potentially leading to misrepresentations or omissions of clinically relevant features. Furthermore, while theoretically feasible for quality assessment, traditional reader studies by radiologists face practical limitations due to their time-consuming nature and the variability of readers [5]. Hallucinations are uncommon, so adequately analysing their effect requires large reader studies, rendering them infeasible in common daily testing practice.

We propose to use currently available diagnostic AI to assess the impact on diagnostic accuracy. By employing DL detection models (DLDetect), we aim to develop a diagnostic quality metric (DiagQM) more aligned with clinical relevance than traditional ImagQMs. This DiagQM addresses the key limitations of reader studies by efficiently handling large datasets and minimising reader variability, while also providing a quantifiable measure of hallucinatory effects. We hypothesise that our DLDetect models can discern decreases in diagnostic accuracy caused by hallucinations. In addition, in case there are negative diagnostic implications of DLRecon, as indicated by the reduced diagnostic performance of DLDetect models, we hypothesise a radiologist will validate these diagnostic inconsistencies.

## Materials and methods

### Datasets

This study, approved by an institutional review board, waived the need for informed consent. It involved retrospective data from three medical centres: University Medical Centre Groningen (UMCG, Groningen, The Netherlands) between 2014 and 2020, Martini Hospital Groningen (MHG, Groningen, The Netherlands) between 2013 and 2020, and Radboud University Medical Centre (RUMC, Nijmegen, The Netherlands) in the year 2016. All patients were suspected of having clinically significant prostate cancer (csPCa) based on criteria such as suspicious digital rectal examinations, lower urinary tract symptoms, or elevated prostate-specific antigen levels.

The collected data comprises either biparametric or multiparametric prostate MRI examinations obtained for routine clinical care. The biparametric protocol included a Turbo Spin Echo T2-weighted (T2W) sequence (see Supplementary Material 1, Table 1), high *b*-value (≥ 1400 s/mm^2^) diffusion-weighted imaging, and an apparent diffusion coefficient map. The study comprised 357, 572, and 846 patients from the UMCG, MHG, and RUMC cohorts, respectively. Exclusions were made for studies that did not follow the PI-RADSv2 [6] MRI protocol requirements or for patients with prior prostate treatment. The external RUMC dataset was subjected to the same exclusion criteria before data transfer. After exclusions, the final sample included 1535 patients, of which 733 (~48%) had PI-RADS 4 or higher, comprising 305 from UMCG, 384 from MHG, and 846 from RUMC.

This study focused on T2W sequences for DLRecon-based prostate MRI for specific reasons. T2W imaging is the dominant sequence for transitional zone lesions and is crucial for morphological assessment, lesion localisation, and guiding procedures like biopsies. By concentrating on T2W sequences, we aim to assess DLRecon’s impact on anatomical representation without functional data from diffusion-weighted imaging, which PI-RADS designates as the primary sequence for peripheral lesions.

Expert uroradiologists with 4–25 years of experience assigned PI-RADS scores and delineated all (likely) csPCa lesions (defined as a PI-RADS score ≥ 4), serving as the reference standard [7–9].

### Retrospective undersampling in K-space

T2W acquisitions (DICOMs) were retrospectively undersampled. The slices were converted to the frequency domain, commonly referred to as k-space, using a fast Fourier transform (FFT). The k-space acquisition is known to be time-intensive, especially during the acquisition of phase-encoding lines. An approach to accelerate the acquisition is to acquire fewer phase-encoding lines, leading to aliasing artefacts in reconstructed images. During the processing of raw k-space data, zero-filling is commonly applied before conversion into an image to achieve higher spatial resolution and a smoother appearance. To take the zero-filling into account, we utilised a binary mask to capture the k-space regions that were acquired. Following this, zero-padding was added to the binary mask in a manner analogous to the zero-filling typically applied to acquired k-space data. Finally, the reconstruction from the undersampled data uses the inverse of the FFT shift, followed by the inverse fast Fourier transform (IFFT), to convert the masked k-space back to the image domain.

**Fig. 1:**
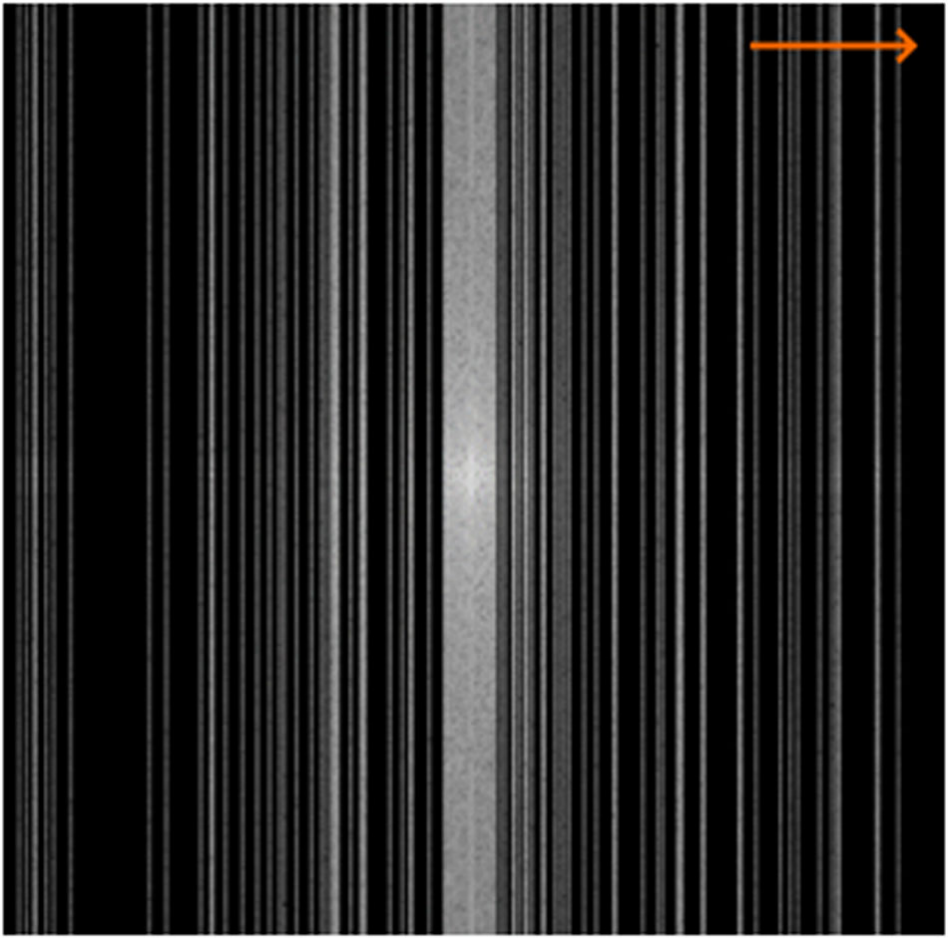
Illustrating a 512x512 2D binary k-space mask with R8 undersampling, employing a 20% central sampling approach. The phase-encoding direction, which is row-based in our multicentre dataset, was undersampled using an exponential random distribution for peripheral k-space lines. Consequently, blurring in the horizontal direction is observed, indicated by the orange arrow

The k-space undersampling mask was designed to subsample the row-based phase-encoding (horizontal) direction (Fig. 1), making it compatible with the multicentre dataset. Research has shown that fully sampling the centre of k-space, which corresponds to low spatial frequencies, improves the signal-to-noise ratio and yields favourable ImagQMs [10]. On the other hand, sampling in the periphery of k-space helps preserve high-resolution details and sharp edges, essential for representing small anatomical structures. We used a 20% central and 80% peripheral k-space undersampling scheme for optimal balance. In practical terms, for a 4-fold (R4) undersampling where 25% of k-space is sampled, 20% of the samples would be drawn from the centre and the remaining 80% from the periphery. This approach aimed to maximise diagnostic utility by enabling the detection of small-sized lesions characterised by high-frequency information while maintaining overall image quality.

### The reconstruction model

For the DLRecon model, we adopted the 3D U-Net [11] architecture, which is widely used for image-based data (see Supplementary Material 2, Fig. 1a). The dataset contains 1535 DICOM images, each with its corresponding PI-RADS segmentation. Due to the absence of multicoil k-space data, we used simulated k-space to carry out image space-based reconstruction tailored for lesion detection algorithms. Therefore, employing an image space-based U-Net is driven by the need to compute a DiagQM through the performance of a subsequent csPCa DLDetect model.

The DLRecon model inputs a centre-cropped, undersampled, and aliased image. The objective is to recover lost details and eliminate aliasing artefacts from k-space undersampling. The training is performed on 4- and 8-fold (R4 and R8) subsampling rates. Specific training parameters include SSIM for the loss function, a batch size of 12, and an Adam optimiser with a 4e–4 learning rate. Additionally, early stopping was used to reduce the risk of overfitting. During training, data augmentation included rotations (25% probability, −30 to 30 degrees), the addition of normally distributed noise (60% probability, 0–0.003 range), and mirroring (50% probability). Training completed within 48 hours on a 32-GB Tesla V100 GPU using the Keras software libraries. The code is available on GitHub (Repo: ProstateMRI-DLReconVsDiagMetrics).

The data for the DLRecon model is preprocessed in four steps: k-space synthesis combined with undersampling, resampling, centre cropping, and normalisation. First, the image is undersampled with the binary subsampling mask. Next, the image undergoes resampling using nearest neighbour interpolation to achieve 0.5 × 0.5 mm voxel spacing, leaving the z-direction unchanged. Subsequently, the image dimensions are standardised through centre cropping (256, 256, 16). Lastly, instance-wise min/max normalisation (scaled between 0 and 1) ensures uniform pixel values, improving model generalisation.

### The detection model

**Fig. 2:**
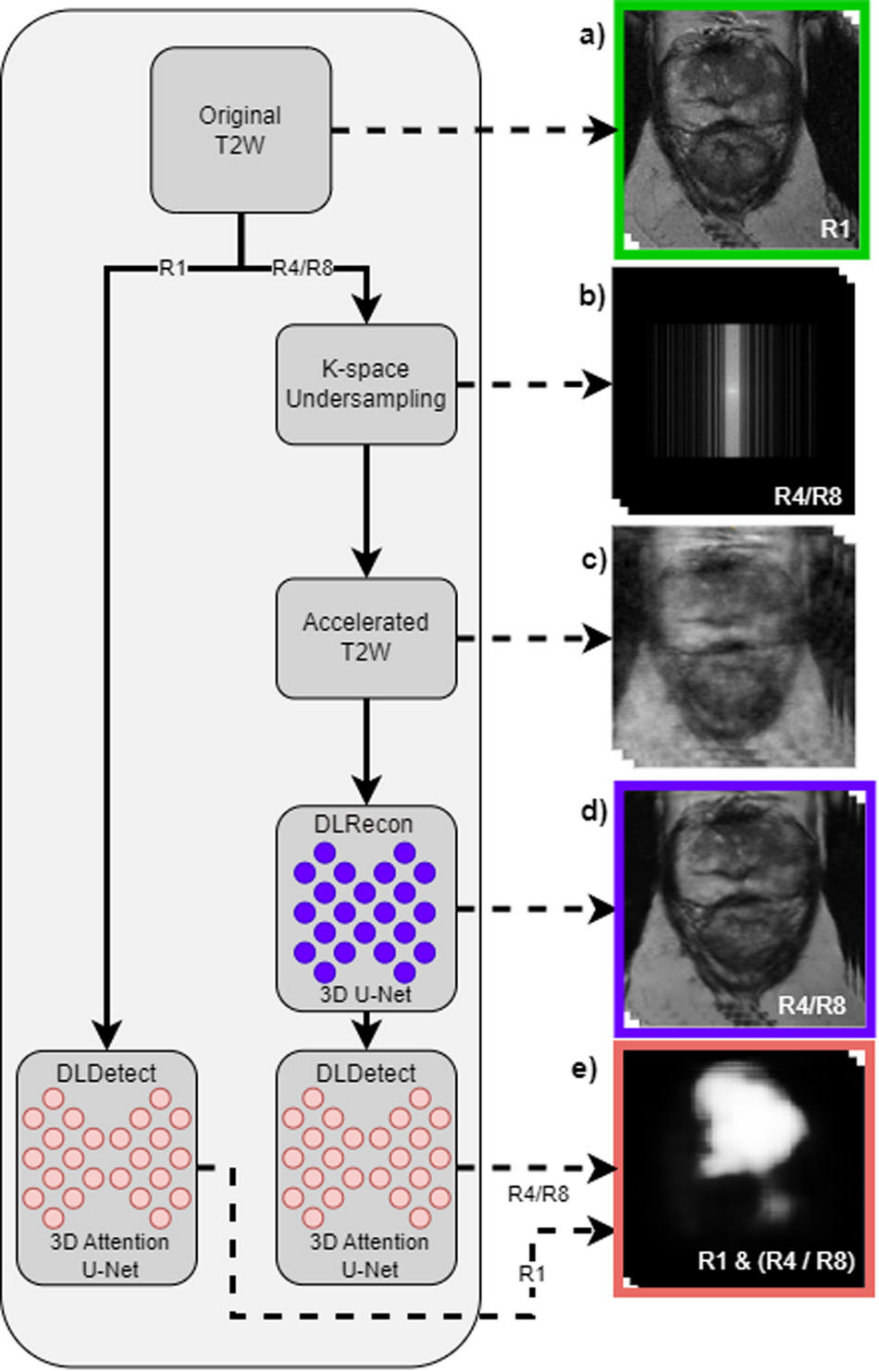
Overview of the Prostate MRI Reconstruction and likely csPCa Detection Pipeline. The workflow begins with the original T2W MRI (**a**), which either proceeds unchanged to the csPCa detection model or undergoes k-space undersampling at accelerations R4/R8 (**b**), resulting in an accelerated image (**c**). Following DL reconstruction (DLRecon) via a 3D U-Net, the improved image (**d**) is run through a DL detection (DLDetect) model to assign csPCa likelihood to each voxel (**e**). The SSIM compares images (**a**) and (**d**) visually, while diagnostic accuracy for likely csPCa is assessed using (**e**) across accelerations R1/R4/R8, aided by PI-RADS annotations from a radiologist

To evaluate the clinical utility of DL reconstructions, we deployed a specialised 3D U-Net [9], distinct from the reconstruction model, solely focused on locating likely csPCa (Fig. 2) in T2W scans (see Supplementary Material 2, Fig. 1c for model architecture). The model has an attention mechanism and squeeze-and-excitation layer to improve feature learning [9, 12]. Specifically, the model was tailored for T2W scans to exclude diffusion-weighted imaging, apparent diffusion coefficient, and dynamic contrast-enhanced sequences, ensuring an unbiased evaluation of the diagnostic quality of T2W sequences. The model generates a 3D heatmap, assigning each voxel a likelihood of csPCa. The training employed a weighted binary cross-entropy loss function. Class weights were set to 0.05 and 0.95 for the negative and positive classes, respectively, to address the class imbalance and to prioritise lesion features over the background tissue. We employed 5-fold cross-validation on 80% of the dataset, reserving 20% as a test set. The highest-performing models from each validation fold were stored. These models were used to evaluate the diagnostic quality of DL-reconstructed T2W MRI scans in the test set. In our methodology, we chose not to retrain the DLDetect models for each acceleration, reflecting the reality in clinical practice where radiologists do not undergo specific retraining for each new technique. This approach prevents the models from normalising hallucination effects—false positives or negatives due to reconstruction—which could mask true diagnostic accuracy.

Preprocessing data for the diagnostic DLDetect models involved resampling, centre cropping, and z-normalisation. Images were resampled to a consistent voxel spacing (0.5, 0.5, 3.0) mm across the multicentre dataset, with a crop size of (180, 180, 16) to focus on the prostate. A larger crop was used in the reconstruction model as the additional field of view could enhance its performance. Instance-wise z-score normalisation standardised voxel values to a mean of zero and standard deviation of 1. Data augmentation included a 10% probability of rotation (−30 to 30 degrees), a 30% probability of adding normally distributed noise (0–0.001 multiplier), and a 50% probability of horizontal flipping.

### Experiments

Our study involved two experiments that evaluated the impact of DLRecon on visual and diagnostic quality using ImagQMs and a DiagQM. See Fig. 2 for a flowchart of the reconstruction and evaluation pipelines. The first experiment employed a Wilcoxon test to statistically assess the visual quality of reconstructions, focusing on SSIM as the ImagQM. The SSIM ranges from −1 to 1, with 1 indicating identical visual quality to the reference image and lower values indicating decreased quality. We analysed reconstruction quality recovery on two subsampling factors, R4 and R8, using both naïve and U-Net-based reconstruction methods. Here, naïve reconstruction refers to inversion from subsampled k-space.

The second experiment evaluated how effectively diagnostic DLDetect models could recognise likely csPCa in reconstructed MR images. Diagnostic accuracy was measured using free-response operating characteristic (FROC) analysis, a methodology used to evaluate a system’s lesion detection sensitivity alongside the false-positive rate, as a patient can have multiple lesions. Within this framework, the diagnostic accuracy metric used was the partial area under the curve (pAUC) of the FROC curve. Specifically, the study employed pAUC values ranging between 0.1 and 2.5 false positives per patient as the DiagQM.

A permutation test compared the pAUC between R4 and R8 subsampling factors across both reconstruction methods. With true positives requiring a 10% overlap with ground truth lesions [9, 13], we aimed to demonstrate non-inferior diagnostic performance (DiagQM) and visual quality (ImagQMs) compared to fully sampled MRI (R1). From the 1535 scans, we designated 80% (*N* = 1229) exclusively for training and validation, employing five-fold cross-validation. The remaining 20% (*N* = 306) constituted a different unseen separate held-out test set for final evaluation. This data-splitting method was consistently applied for the DL reconstruction and detection models. In addition to these quantitative metrics, we also qualitatively investigated the manifestation and impact of hallucinatory effects in the reconstructed images.

### Hallucination validation

We conducted a small reader study to validate the DLDetect as a proxy for assessing the diagnostic quality of DLRecon. We selected a balanced set of 30 cases from our test set based on their DLetect observed differences in csPCa likelihood. Comprising 15 cases with the highest difference in PCa detection (the ‘Inconsistent Set’) and 15 with the lowest difference (the ‘Consistent Set’).

An experienced radiologist (> 8 years) reviewed R1 vs. R4/R8 image pairs, categorising them as consistent, showing minor variation, or inconsistent. Cohen’s kappa analysis compared radiologist’s and DLDetect’s ability to agree on observed inconsistencies (hallucinations). For a detailed explanation of the study’s methodology and findings, please refer to Supplementary Materials 3.

## Results

**Fig. 3:**
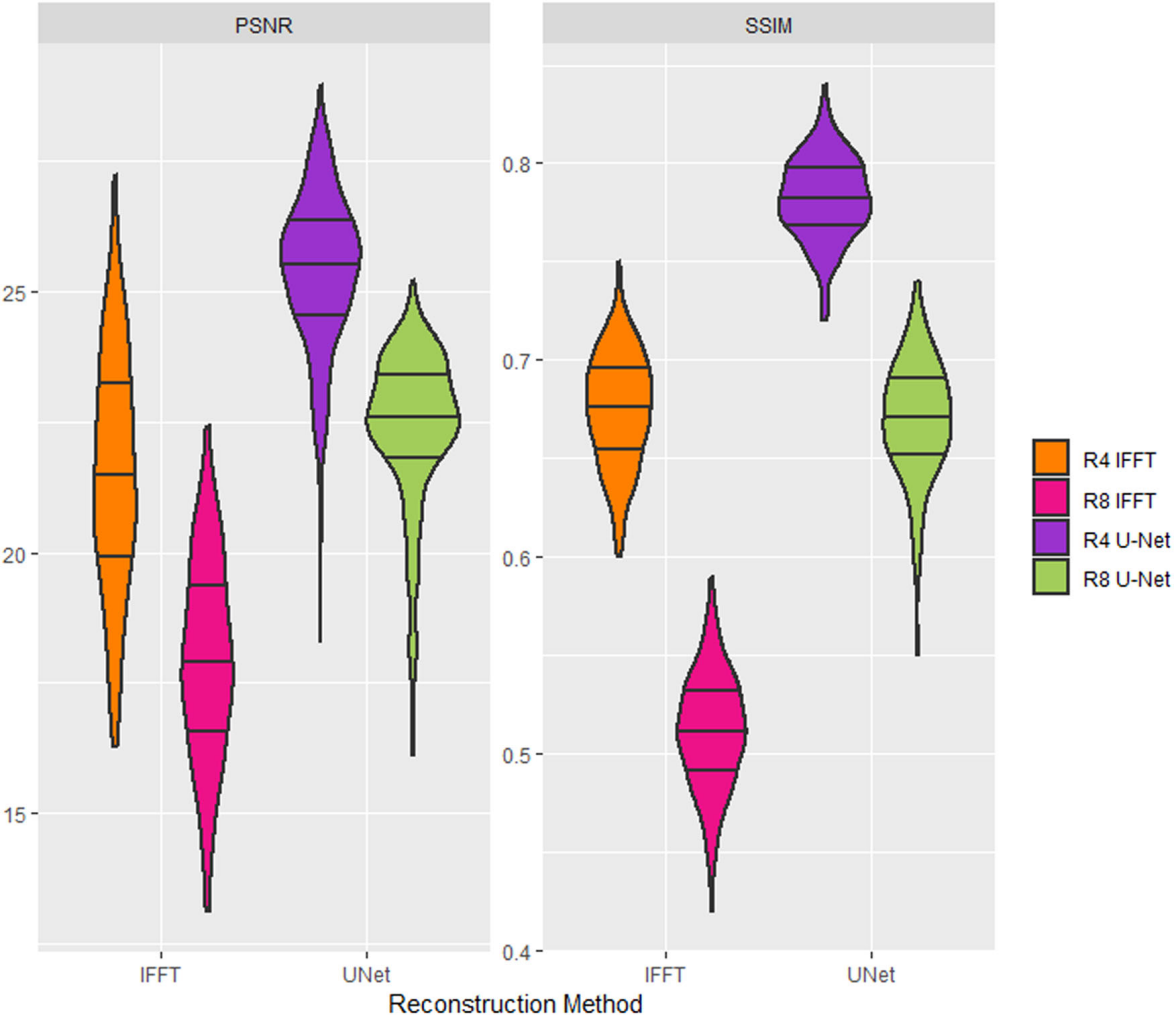
Violin plot comparing ImagQMs SSIM and PSNR across 306 test cases. This showcases the performance of two reconstruction methods: naïve (IFFT) and DLRecon (U-Net) methods at subsampling factors R4 and R8

The first experiment (Fig. 3) demonstrated that DLRecon significantly improved the visual quality compared to the naïve (IFFT) reconstruction on the test set (*N* = 306). Specifically, SSIM values improved in 4-fold subsampled data from IFFT data: 0.78 ± 0.02 vs. 0.68 ± 0.03 (*p* < 0.001), and for R8 data: 0.67 ± 0.03 vs. 0.51 ± 0.03 (*p* < 0.001).

**Fig. 4:**
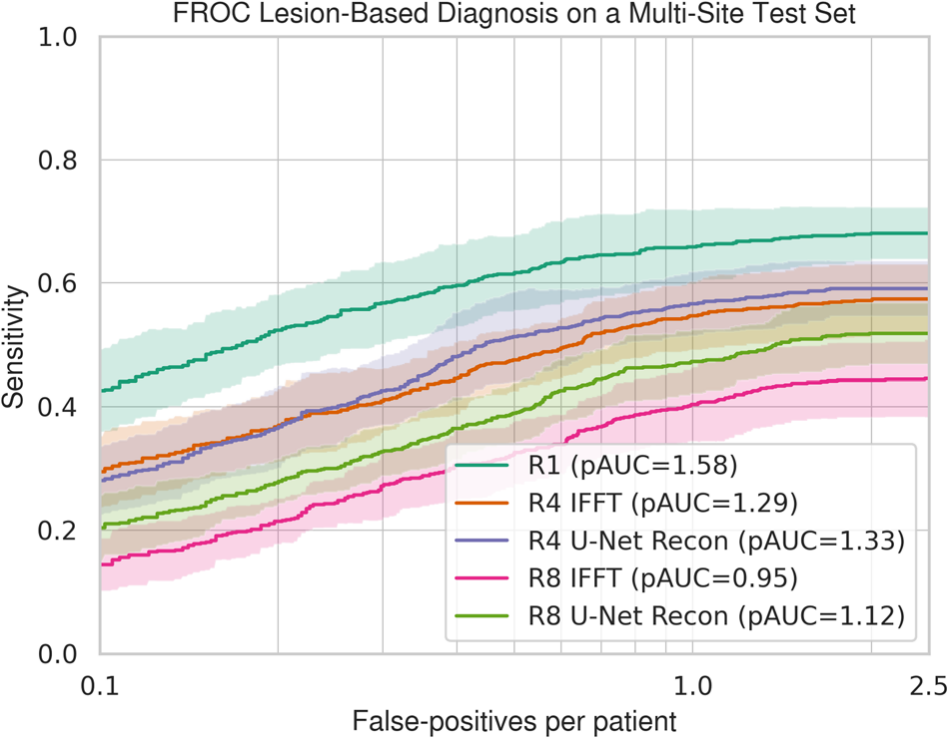
FROC curves for likely csPCa detection performance. This figure presents FROC curves for DLRecon using U-Net and IFFT methods. The curves are plotted on a logarithmic scale to depict false-positive lesions per patient and include 95% confidence intervals. Data is derived from a multisite test set of 306 cases. The legend includes pAUC values, serving as the DiagQM, with R1 as the baseline detection AI trained on fully sampled MRI

The second experiment (Fig. 4) revealed diminished diagnostic performance in R4 and R8 reconstructions compared to fully sampled R1 data of the test set (*N* = 306). Specifically, pAUC values for R4 were 1.29 (CI 1.23–1.35) for naïve and 1.33 (CI 1.28–1.39) for U-Net, both significantly lower than the 1.58 (CI 1.52–1.64) for R1 data. Permutation tests confirmed these differences (naïve *p* = 0.02, U-Net *p* = 0.024). A similar pattern was observed for R8.

We further assessed the diagnostic performance of DLDetect models in spotting likely csPCa using both DLRecon and naïve reconstructions. As shown in Fig. 4, no significant disparities were observed between these methods in either R4 (pAUC 1.33 [CI 1.28–1.39] vs. 1.29 [CI 1.23–1.35], *p* = 0.37) or R8 (pAUC 1.12 [CI 1.07–1.17] vs. 0.95 [CI 0.89–1.01], *p* = 0.067). Nonetheless, a sensitivity reduction of at least 10% was observed compared to fully sampled R1 data.

In addition to a lesion-based analysis, a patient-based receiver operating characteristic analysis of our test set revealed AUC values of 0.87 for R1 and 0.78 for R4. Using an optimal threshold of 0.76, we found sensitivity for R1 and R4 to be 0.84. Specificity decreased in R4 (0.54) compared to R1 (0.75), causing the positive predictive value to drop from 0.74 in R1 to 0.61 in R4. The negative predictive value decreased from 0.85 to 0.81. These findings highlight a trade-off in DL reconstructions, maintaining sensitivity but with lower specificity.

**Fig. 5:**
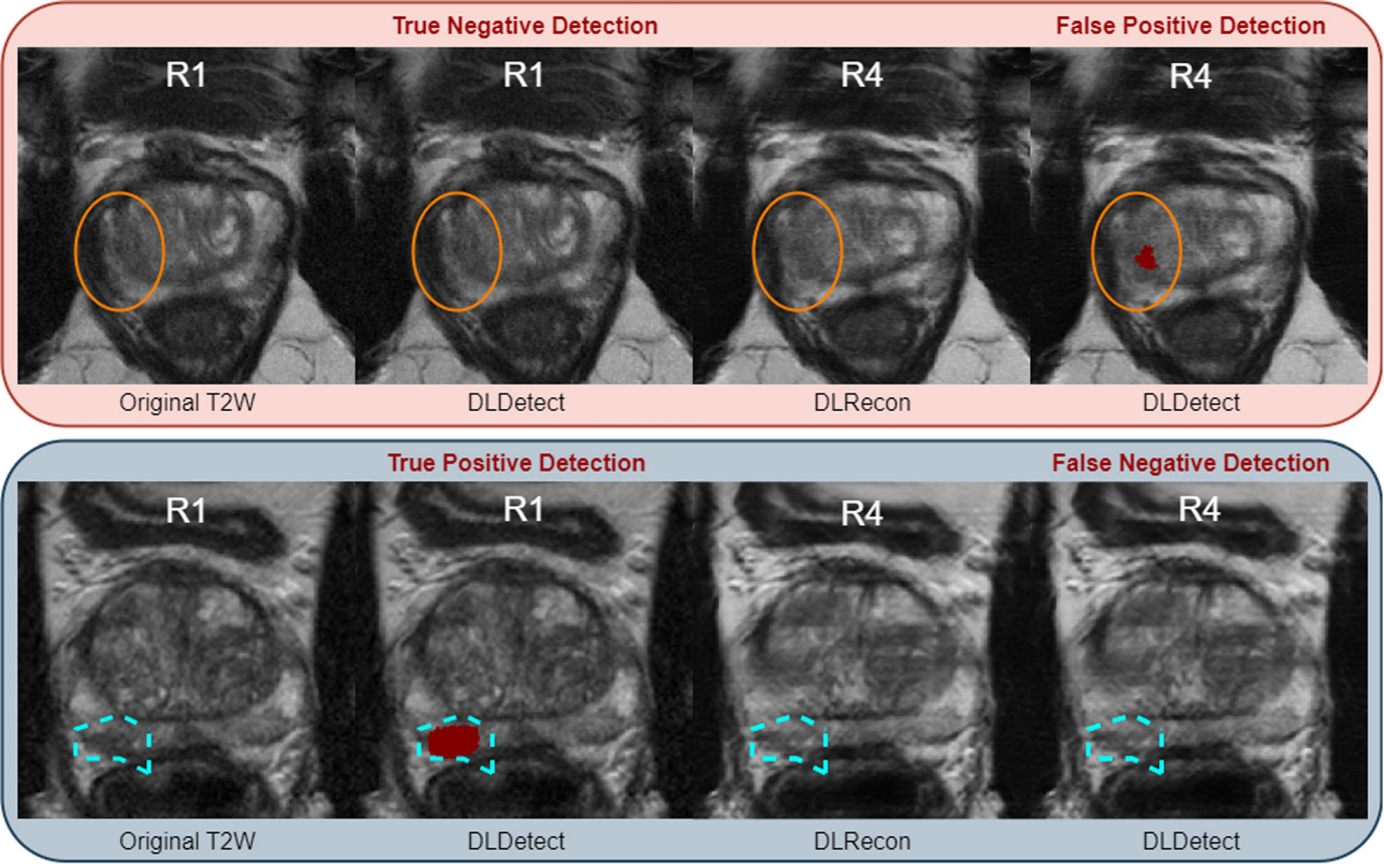
Demonstrating hallucinatory effects in DL reconstruction (DLRecon). The top row highlights a false-positive detection, where the detection model (DLDetect) incorrectly identifies a lesion in the R4 DLRecon—marked by the red overlay—which was not present in the original unaccelerated (R1) image, indicating a hallucination effect that introduces a new lesion post-reconstruction. The bottom row presents a false negative case: a lesion detected by DLDetect in the original R1 image is not identified in the R4 reconstructed image, illustrating a hallucination effect where a true lesion is obscured following the reconstruction process

Figure 5 (top row) provides an example of a hallucinatory effect induced by the DLRecon. In this specific instance, the hallucination led the DLDetect model to predict a lesion not present in the corresponding fully sampled R1 image. The reconstructed image displayed a subtle discontinuity in peripheral zone intensity (marked by the orange circle), a characteristic commonly linked to transition zone lesions. These findings underscore the risk of ‘hallucination effects’ influencing diagnostic models, leading to false-positive or false-negative detections and reduced model sensitivity while producing visually appealing images.

In the reader study, Cohen’s kappa values indicated a low to moderate agreement between the radiologist’s evaluations and the DLDetect models, with a kappa of 0.2 (CI: −0.097 to 0.5) for R4 and 0.4 (CI: 0.079 to 0.72) for R8. These findings are visually represented in Supplementary Materials 3, Fig. 1.

## Discussion

In this study, we developed a novel evaluation methodology for the diagnostic effectiveness of DLRecon techniques in MRI that can be effortlessly scaled to accommodate large datasets. We incorporated diagnostic AI into the assessment method, creating a benchmark (DiagQM) to assess the diagnostic reliability alongside the conventional ImagQMs, like the SSIM and PSNR. Specifically, we found that while conventional ImagQMs may yield visually appealing results, the diagnostic accuracy seems affected, especially at higher subsampling rates. This limitation is exemplified by a DL hallucination that led to a false-positive detection (Fig. 5).

The reader study revealed a moderate alignment between radiologists’ evaluations and DLDetect models in identifying diagnostic inconsistencies introduced by DLRecon. The concordance suggests that detection models could enhance DLRecon evaluation methods. However, while promising, these findings also underscore the indispensable role of radiologists in validating AI interpretations, particularly in complex diagnostic situations requiring nuanced judgment. As hypothesised, evaluating DLRecon with DLDetect models yielded meaningful insights into the diagnostic performance of DLRecon models in contrast to conventional ImagQMs. The disparity between ImagQMs and DiagQMs underscores the necessity to shift from purely visual evaluation metrics to those that matter for clinical decision-making. In this context, DiagQMs serve as an example, emphasising the importance of evaluating a DLRecon model’s capability to display pathological features accurately.

In addition to the results presented in this study, it is worth noting that existing, state-of-the-art DLRecon models [14–17] rely heavily on ImagQMs (e.g., SSIM), which, as demonstrated in our study, may not be the most appropriate approach. The anticipated influx of DLRecon models from various vendors for clinical practice in the near future underscores the heightened significance of recognising the limitations associated with visual metrics. In addition to offering more meaningful insights, DiagQMs enable rapid throughput to examine large datasets. Our proposed assessment method, using DLDetect models, required less than 30 minutes of computing time to evaluate a total of 918 cases across three acceleration factors (R1, R4 and R8). In contrast, a radiologist would require significantly more time, at least up to 2–3 weeks, to perform a similar assessment.

Our study resonates with the findings of the fastMRI challenge, which applied DLRecon models to subsampled k-space brain data [4]. In this challenge, the primary evaluation metric was SSIM, while a secondary assessment was performed by radiologists using a 5-point Likert scale to rate visual quality. Although SSIM scores typically correlated with positive radiologist evaluations, the radiologists observed hallucination effects, where DLRecon models altered specific brain abnormalities to mimic normal structures. Unlike the fastMRI challenge, which relies on incidental findings by radiologists, our methodology introduces a quantifiable metric for diagnostic accuracy, making our approach well-suited for large datasets.

Our study had certain limitations. First, we relied on retrospective k-space subsampling from DICOMs, a method that utilises image space data rather than multicoil k-space data. Consequently, the results of this study should be validated in future research using multicoil k-space data. It is worth noting that the use of image space data is an accepted approach in many studies [18–23] due to the limited availability of raw k-space data.

Secondly, our study was limited to T2W sequences, omitting functional imaging, such as diffusion-weighted imaging, essential for comprehensive PCa evaluation. Future work should include these modalities in DL models for reconstruction and detection. Thirdly, the reader study agreement was moderate. The side-by-side comparison by a reader is different in some cases from the independent AI assessments. However, in retrospect, AI identified reconstruction hallucinations and showed image degradation that was supportive of diagnostic differences.

To the best of our knowledge, despite the abundance of high-performing and well-validated DLDetect models available [24, 25], none have been used to evaluate the diagnostic accuracy of DLRecon models, as was the case in this study. These DLDetect models are inherently designed to streamline diagnostic tasks into a single, quantifiable quality metric, making them well-suited for evaluating DLRecon methods. Future research could consider a more balanced approach that combines the DiagQM with human experts, ensuring that both components continue to play integral roles in the diagnostic process. This synergy between automated diagnostic metrics and the expertise of human radiologists holds the potential to further enhance the accuracy and reliability of medical image reconstructions and interpretations.

## Conclusion

DL reconstruction produces visually appealing images but may reduce diagnostic accuracy. Incorporating diagnostic AI into the assessment framework offers a clinically relevant metric essential for adopting reconstruction models into clinical practice.

## Supplementary information

Electronic Supplementary Material

## Abbreviations

AI: Artificial intelligence
csPCa: Clinically significant prostate cancer
DiagQM: Diagnostic quality metric
DL: Deep learning
DLDetect: Deep learning detection
DLRecon: Deep learning reconstruction
FFT: Fast Fourier transform
FROC: Free-response operating characteristic
IFFT: Inverse fast Fourier transform
ImagQM: Image quality metric
pAUC: Partial area under the curve
PCa: Prostate cancer
PSNR: Peak signal-to-noise-ratio
SSIM: Structural similarity index measure
T2W: T2-weighted
