# Supplementary material for: Assessing deep learning reconstruction for faster prostate MRI: visual vs. diagnostic performance metrics
Source: Eur Radiol. 2024 May 9;34(11):7364–72. doi: 10.1007/s00330-024-10771-y (PMC11519109; doi:10.1007/s00330-024-10771-y)
Supplement: Supplementary file 1 — Electronic Supplementary Material [file 330_2024_10771_MOESM1_ESM.pdf]

Assessing Deep Learning Reconstruction for Faster Prostate MRI: Visual vs. Diagnostic Performance Metrics

Electronic Supplementary Material (ESM)

Supplementary materials

Supplementary materials 1 2

Overview of MRI Characteristics 2

Supplementary materials 2 3

Deep Learning Model Architectures 3

Supplementary materials 3 4

Reader Study Materials and Methods 4

Supplementary materials 1

Overview of MRI Characteristics

Continuous values are presented as mean ± standard deviation.

	University Medical Center Groningen (UMCG)	*Martini Hospital Groningen (MHG)*	*Radboud University Medical Center (RUMC)*
Dataset	20%	23%	57%
Scanner Models	Achieva* 33%, Ingenia* 3%, Aera** 11%, Avanto** 10%, Espree** 1%, Prisma** 17%, Skyra** 24%	Achieva dStream* <1%, Ingenia* 95%, Intera* 5%	Prisma_fit** 8%, Skyra** 92%, TrioTim** <1%
In-plane Resolution (mm)	0.43 ± 0.02	0.35 ± 0.00	00.51 ± 0.01
Slice thickness (mm)	3.04 ± 0.04	3.05 ± 0.05	3.02 ± 0.08
Spacing between slices (mm)	3.23 ± 0.08	3.05 ± 0.05	3.60 ± 0.07
Number of averages	3.17 ± 0.07	1.05 ± 0.09	3.96 ± 0.64
Echo Train Length	25.22 ± 1.76	20.27 ± 0.94	25.00 ± 0.00
Field of View (mm)	186 x 186 (± 16)	348 x 348 (± 11)	194 x 194 (± 21)

Table 1 Characteristics of T2W transversal MRI from UMCG, MHG, and RUMC.
*: Philips Medical Systems, Best, The Netherlands, **Siemens Healthineers, Erlangen Germany.

Supplementary materials 2

Deep Learning Model Architectures

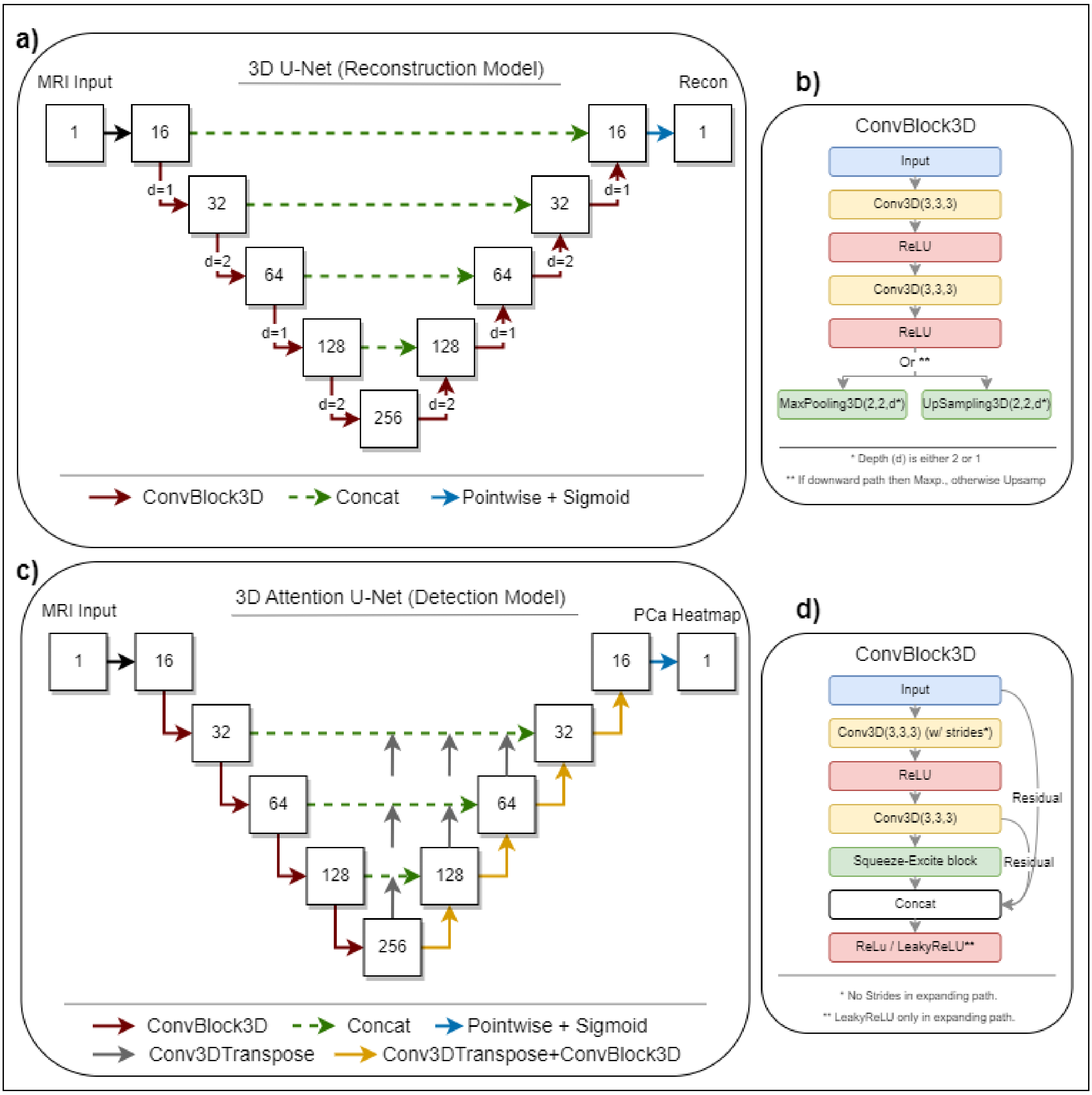

Fig 1 Schematic representations of MRI reconstruction and detection model. **(a)** shows the 3D U-Net model[1] structure for image reconstruction. **(b)** outlines the components of a ConvBlock3D of the reconstruction model. **(c)** presents the 3D Attention U-Net model[2] used for lesion detection, and **(d)** details the ConvBlock3D with attention mechanism for the detection model.

---

[1]Yin XX, Sun L, Fu Y, et al (2022) U-Net-Based Medical Image Segmentation. J Healthc Eng 2022
[2]Saha, A., Hosseinzadeh, M., & Huisman, H. (2021). End-to-end prostate cancer detection in bpMRI via 3D CNNs: Effects of attention mechanisms, clinical priori and decoupled false positive reduction. *Medical image analysis*, *73*, 102155. https://doi.org/10.1016/j.media.2021.102155

Supplementary materials 3

Reader Study Materials and Methods

For this study, 30 cases from the test set were selected, 15 with the smallest and 15 with the largest discrepancies in diagnostic predictions made by the DLDetect model. The cases were chosen based on the variance in predicted likelihoods for csPCa between the original (R1) and the accelerated reconstructed images (R4 or R8). These variances are indicative of potential diagnostic alterations attributable to hallucinatory artefacts introduced during the DL reconstruction process.

A change in the estimated likelihood of csPCa at the patient level determines an 'inconsistent' comparison between an original and a DLRecon image. For example, if an unaccelerated image shows a 0.2 likelihood of csPCa, but its reconstructed version shows a 0.80 likelihood, and the patient's overall diagnosis is 'negative', this represents an inconsistent diagnosis. This inconsistency arises because the reconstruction shifts the case from a probable negative to a false positive result.

A radiologist was tasked with assessing pairs of MR images to determine if diagnostic decisions based on R4 or R8 reconstructions align with those from R1 images, focusing on the consistency of diagnostic features rather than image quality. The radiologist was informed that the set includes 15 cases likely to contain hallucinations and 15 unlikely, without knowledge of their specific classifications. Each evaluation involves a two-step process: first, examining the accelerated (R4 or R8) image to form an initial diagnostic impression, followed by reviewing the corresponding unaccelerated (R1) image. The radiologist then categorizes the case into one of three diagnostic outcomes: consistent diagnosis, minor diagnostic variation, or inconsistent diagnosis. The cases were presented in a randomized order.

The 3-tier scoring system:

1. Diagnostic Consistency: No meaningful differences. Similar diagnostic interpretation.
2. Minor Diagnostic Variation: Minor differences possibly affecting diagnostic interpretation.
   (e.g. Pirads 2 on the accelerated images would become Pirads 1 on the unaccelerated images).
3. Diagnostic Inconsistency: Clear differences affecting diagnostic interpretation.

We implemented a 'Minor Diagnostic Variation' tier within our three-tier scoring system to accommodate the radiologist's diagnostic certainty. For binary statistical analysis, we classified level-1 scores as 'consistent' and combined level-2 and level-3 scores as 'inconsistent,' allowing us to distinguish between cases with diagnostic discrepancies and those without.

The analysis focused on using Cohen's kappa to measure the level of agreement between the radiologist's evaluations and the AI-detected differences in diagnoses. Two kappa calculations were performed: one compared the results for images reconstructed at R4 acceleration and the other at R8 acceleration against the standard R1 images.

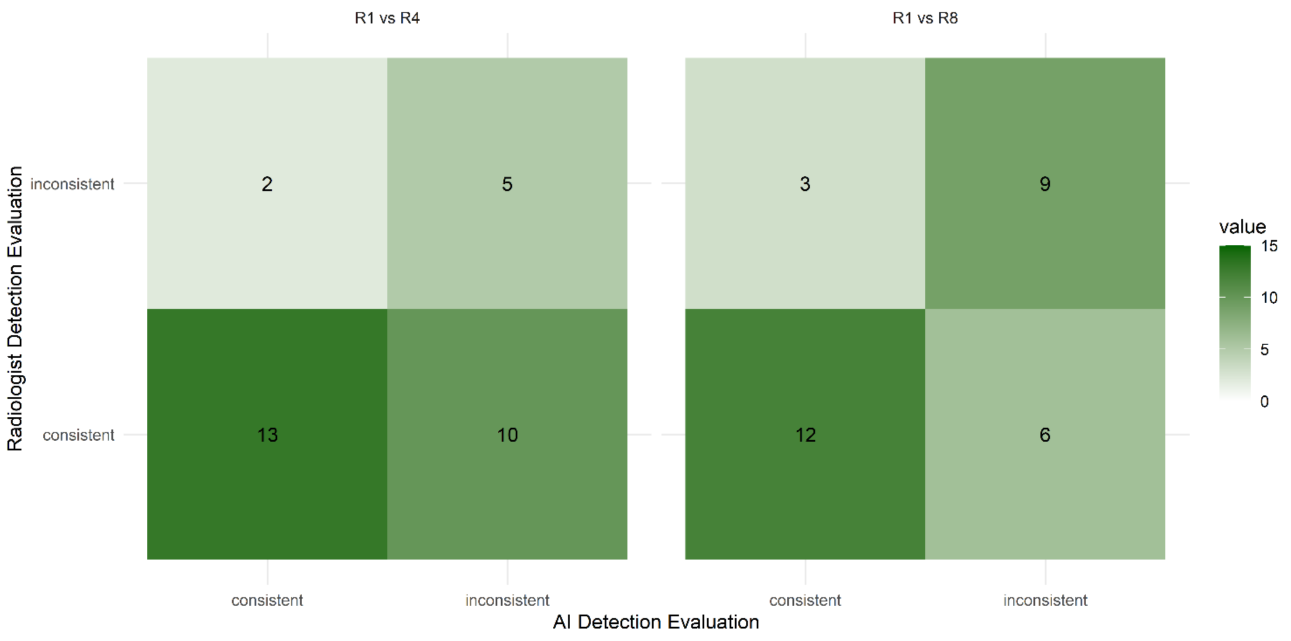

Fig 2 Agreement between Radiologist and AI Detection Evaluations for R1 vs R4 and R1 vs R8 Image Sets. The matrix displays the count of cases where the radiologist's evaluation is consistent (bottom row) or inconsistent (top row) with the AI detection model's evaluation (left and right columns). Darker shades indicate a higher number of cases. The left matrix compares R1 with R4 reconstructions, and the right matrix compares R1 with R8 reconstructions.

Supplementary materials 4

CLAIM: Checklist for Artificial Intelligencer in Medical Imaging

This section contains the CLAIM[3] checklist, finalised through consensus between two authors. Our responses are organised into four categories: 'Reported,' 'Not Reported,' 'Not Applicable,' and 'Not Explicit.' This organisation aims to succinctly showcase the extent to which our study adheres to the recommended practices for AI research in medical imaging.

Section / Topic	No.	Item	
TITLE / ABSTRACT			
	**1**	Identification as a study of AI methodology, specifying the category of technology used (e.g., deep learning)	**Reported**
	**2**	Structured summary of study design, methods, results, and conclusions	**Reported**
INTRODUCTION			

[3] Mongan J, Moy L, Kahn CE Jr. Checklist for Artificial Intelligence in Medical Imaging (CLAIM): a guide for authors and reviewers. Radiol Artif Intell 2020; 2(2):e200029. https://doi.org/10.1148/ryai.2020200029

	**3**	Scientific and clinical background, including the intended use and clinical role of the AI approach	**Reported**
	**4**	Study objectives and hypotheses	**Reported**
METHODS			
Study Design	**5**	Prospective or retrospective study	**Reported**
	**6**	Study goal, such as model creation, exploratory study, feasibility study, non-inferiority trial	**Not Explicit**
Data	**7**	Data sources	**Reported**
	**8**	Eligibility criteria: how, where, and when potentially eligible participants or studies were identified (e.g., symptoms, results from previous tests, inclusion in registry, patient-care setting, location, dates)	**Reported**
	**9**	Data preprocessing steps	**Reported**
	**10**	Selection of data subsets, if applicable	**Reported**
	**11**	Definitions of data elements, with references to Common Data Elements	**Not Applicable**
	**12**	De-identification methods	**Not Reported**
	**13**	How missing data were handled	**Not Applicable**
Ground Truth	**14**	Definition of ground truth reference standard, in sufficient detail to allow replication	**Reported**
	**15**	Rationale for choosing the reference standard (if alternatives exist)	**Reported**
	**16**	Source of ground-truth annotations; qualifications and preparation of annotators	**Reported**
	**17**	Annotation tools	**Not Reported**
	**18**	Measurement of inter- and intrarater variability; methods to mitigate variability and/or resolve discrepancies	**Not Applicable**
Data Partitions	**19**	Intended sample size and how it was determined	**Not Applicable**
	**20**	How data were assigned to partitions; specify proportions	**Reported**
	**21**	Level at which partitions are disjoint (e.g., image, study, patient, institution)	**Not Explicit**

Model	**22**	Detailed description of model, including inputs, outputs, all intermediate layers and connections	**Reported**
	**23**	Software libraries, frameworks, and packages	**Reported**
	**24**	Initialization of model parameters (e.g., randomization, transfer learning)	**Reported**
Training	**25**	Details of training approach, including data augmentation, hyperparameters, number of models trained	**Reported**
	**26**	Method of selecting the final model	**Reported**
	**27**	Ensembling techniques, if applicable	**Not Applicable**
Evaluation	**28**	Metrics of model performance	**Reported**
	**29**	Statistical measures of significance and uncertainty (e.g., confidence intervals)	**Reported**
	**30**	Robustness or sensitivity analysis	**Reported**
	**31**	Methods for explainability or interpretability (e.g., saliency maps), and how they were validated	**Reported**
	**32**	Validation or testing on external data	**Not Explicit**
RESULTS			
Data	**33**	Flow of participants or cases, using a diagram to indicate inclusion and exclusion	**Not Reported**
	**34**	Demographic and clinical characteristics of cases in each partition	**Not Reported**
Model performance	**35**	Performance metrics for optimal model(s) on all data partitions	**Reported**
	**36**	Estimates of diagnostic accuracy and their precision (such as 95% confidence intervals)	**Reported**
	**37**	Failure analysis of incorrectly classified cases	**Reported**
DISCUSSION			
	**38**	Study limitations, including potential bias, statistical uncertainty, and generalizability	**Reported**
	**39**	Implications for practice, including the intended use and/or clinical role	**Reported**
OTHER INFORMATION			
	**40**	Registration number and name of registry	**Not Applicable**

	**41**	Where the full study protocol can be accessed	**Reported**
	**42**	Sources of funding and other support; role of funders	**Reported**
